# T Helper Cell Infiltration in Osteoarthritis-Related Knee Pain and Disability

**DOI:** 10.3390/jcm9082423

**Published:** 2020-07-29

**Authors:** Timo Albert Nees, Nils Rosshirt, Jiji Alexander Zhang, Hadrian Platzer, Reza Sorbi, Elena Tripel, Tobias Reiner, Tilman Walker, Marcus Schiltenwolf, Hanns-Martin Lorenz, Theresa Tretter, Babak Moradi, Sébastien Hagmann

**Affiliations:** 1 Clinic for Orthopedics and Trauma Surgery, Center for Orthopedics, Trauma Surgery and Spinal Cord Injury, Heidelberg University Hospital, Schlierbacher Landstr. 200a, 69118 Heidelberg, Germany; timo.nees@med.uni-heidelberg.de (T.A.N.); nils.rosshirt@med.uni-heidelberg.de (N.R.); jijialexander.zhang@med.uni-heidelberg.de (J.A.Z.); hadrian.platzer@med.uni-heidelberg.de (H.P.); reza.sorbi@med.uni-heidelberg.de (R.S.); elena.tripel@med.uni-heidelberg.de (E.T.); tobias.reiner@med.uni-heidelberg.de (T.R.); tilman.walker@med.uni-heidelberg.de (T.W.); marcus.schiltenwolf@med.uni-heidelberg.de (M.S.); babak.moradi@med.uni-heidelberg.de (B.M.); 2 Department of Internal Medicine V, Division of Rheumatology, Heidelberg University Hospital, Im Neuenheimer Feld 410, 69120 Heidelberg, Germany; Hannes.Lorenz@med.uni-heidelberg.de (H.-M.L.); theresa.tretter@med.uni-heidelberg.de (T.T.)

**Keywords:** osteoarthritis, pain, inflammation, synovial fluid, synovial membrane, blood, T cells, infiltration

## Abstract

Despite the growing body of literature demonstrating a crucial role of T helper cell (Th) responses in the pathogenesis of osteoarthritis (OA), only few clinical studies have assessed interactions between Th cells and OA—related symptoms. Yet, the inclusion of clinical data in the interpretation of cellular analyses of Th cell infiltration is essential to reveal the mechanisms underlying the complex pathophysiology of OA pain and disability. Thus, the aim of the study was to analyze the infiltration pattern of Th cells in systemic (peripheral blood) and joint-derived (synovial membrane and fluid) samples from patients with knee OA in relation to OA-induced pain and disability. Therefore, radiographic OA severity, knee pain and function of 47 OA patients undergoing knee arthroplasty were evaluated prior to surgery. In parallel, samples of peripheral blood (PB), synovial membrane (SM) and synovial fluid (SF) were harvested and analyzed for different Th subsets using flow cytometry. According to surface marker expression Th cells (CD3^+^ CD4^+^ CD8^−^) were assigned to the Th subsets Th1 (CXCR3^+^, CCR5^+^), Th2 (CCR3^+^, CCR4^+^) and Th17 (CD161^+^, CCR6^+^). Interestingly, infiltration of the SM with all Th subtypes (Th1, Th2, Th17) significantly correlated with OA-induced disability. Most importantly, synovial CCR5^+^ and CCR3^+^ Th cell infiltration was associated with OA-related knee pain and disability. Furthermore, higher percentage rates of CXCR3^+^ Th cells in all tissue samples (PB, SM, SF) showed significant associations with OA severity. In contrast, increasing percentage rates of CD161^+^ Th cells in SM samples corresponded to a better functional outcome. In conclusion, the current study provides an extensive profile of the Th cell infiltration pattern in PB, SF and SM from patients with clinically relevant knee OA. Th cell infiltration of the SM might play a crucial role not only in the pathogenesis of OA but also in the development of OA-related knee pain and disability.

## 1. Introduction

Over 250 million people worldwide ≥ suffer from clinically relevant pain and disability due to osteoarthritis (OA) [1]. A large number of OA patients present clinical signs of inflammation, such as joint swelling and effusion. Nevertheless, OA has long been interpreted as a non-inflammatory “wear and tear” disease resulting in loss of articular cartilage. Recent findings provide compelling evidence that joint inflammation is a key mediator of OA pathogenesis [2]. Inflammatory mechanisms disrupting the balance between anabolic and catabolic processes of normal cartilage homeostasis lead to progressive joint degeneration in both animal models and humans with OA [3,4,5]. Moreover, the clinical hallmarks of OA-pain and functional disability of the affected joints seem to correlate with synovial inflammation [6,7,8,9,10]. Mononuclear cells (T cells and macrophages) infiltrate the synovial membrane (SM) of OA joints and levels of pro-inflammatory mediators in peripheral blood (PB) and synovial fluid (SF) samples are elevated [11,12,13,14]. Immunohistological and flow cytometry studies have shown that T helper cells (Th; CD3^+^ CD4^+^ CD8^−^, subsequently referred to as CD4^+^; CD = cluster of differentiation) present one of the most prevalent types of activated inflammatory cells in the synovium [12,15,16] and growing evidence indicates a pivotal role of Th cells in the pathogenesis of OA [17]. Analysis of PB and SF samples of OA patients revealed significantly higher levels of CD4^+^ T cells when compared to samples of age-matched healthy controls [18]. Furthermore, the percentage of CD4^+^ cells in SF of OA patients is significantly higher than in matched PB samples [19]. Recently, we have demonstrated that a predominant Th type 1 (Th1) polarization is present in SF samples of patients with end-stage knee OA [14]. Several other studies indicate that both synovial Th1 and Th17 cell infiltration might play an important role in the pathogenesis of OA. In contrast, the pathophysiological role of Th2 responses in OA seems to be limited although further investigations are needed [17].

In summary, convincing evidence indicates that CD4^+^ cells might be key players in the pathogenesis of OA. Especially synovial infiltration of Th subtypes triggering inflammatory responses could be of major importance. Nevertheless, the clinical relevance of synovial and serum Th infiltration remains elusive. Despite the growing body of literature demonstrating a crucial role of Th responses in OA pathogenesis, only few clinical studies have assessed interactions between Th cells and OA-related symptoms. Yet, including clinical data in the interpretation of cellular analyses of Th cell infiltration is essential to reveal the mechanisms underlying the complex pathophysiology of OA pain and disability. Thus, the aim of the current study was—(i) to further characterize the CD4^+^ T cell population in PB, SM and SF samples of knee-OA patients using flow cytometry with different cell surface markers and (ii) to assess associations between Th subtypes and clinical parameters including OA severity, pain and function.

To the best of our knowledge this is the first study analyzing synovial and serum Th1, Th2 and Th17 infiltration in correlation to a broad variety of clinical data of patients suffering from knee OA. In brief, we found that infiltration of the synovial membrane with all Th subtypes (Th1, Th2, Th17) significantly correlates with OA-induced disability. Most importantly, both synovial CD4^+^ CCR5^+^ (CCR = C-C chemokine receptor) and CD4^+^ CCR3^+^ cell infiltration is associated with OA-related knee pain and disability. Furthermore, the percentage of CD4^+^ CXCR3^+^ (C-X-C motif chemokine receptor 3) cells in all tissue samples (PB, SM, SF) shows significant associations with OA severity.

## 2. Experimental Section

### 2.1. Study Population

A total of 47 patients with primary knee OA (35 women, 12 men) consecutively undergoing knee replacement surgery at Heidelberg University Hospital were enrolled in this study. OA was defined according to the American College of Rheumatology criteria. Based on anteroposterior, sagittal and varus-valgus stress radiographs, OA was classified as unicompartmental (UC) or bicompartmental (BC) OA. The Kellgren and Lawrence (K&L) score was used to assess the radiographic severity of OA [20]. Patients with UC OA were scheduled for unicompartmental (UKA) and those with BC OA for total knee arthroplasty (TKA). Underlying inflammatory diseases including RA, clinical and laboratory signs of systemic inflammation, intake of disease-modifying anti-rheumatic drugs (DMARDs) and intra-articular injections of corticosteroids or hyaluronic acid as well as arthroscopy on the target knee within 3 months before enrollment were considered exclusion criteria. The study was conducted in accordance with the local ethics committee of the Medical Faculty at Heidelberg University and the Declaration of Helsinki and was approved by the institutional review board of the Medical Faculty Heidelberg (S333/2007).

### 2.2. Clinical Assessment

To assess the radiographic severity of OA anteroposterior radiographs of the symptomatic knees were used and graded according to the Kellgren and Lawrence (K&L) scoring system (0–IV) [20]. Knee pain and function prior to surgery was assessed using the 11-point (0–10) numerical rating scale (NRS; 0 = no pain; 10 = worst pain), the 12-item self-administered Oxford Knee Score (OKS-12) [21], the American Knee Society score (AKSS) [22] and the Hannover Functional Questionnaire of functional disability caused by OA [23].

### 2.3. Sample Collection

Samples of PB, SM and SF were collected at the time of surgery as previously described [14]. Prior to arthrotomy, needle aspiration was performed to harvest SF. SF samples were stored in sterile tubes for further processing. After arthrotomy SM samples were harvested from the suprapatellar pouch. Concurrently, heparinized PB samples were collected. For overall analyses *n* = 45 PB, *n* = 37 SM and *n* = 24 SF samples were available.

### 2.4. Tissue Processing

Preparation of PB, SM and SF samples for further flow cytometry analysis was performed as previously described [14]. In brief, SF samples were treated with bovine testicular hyaluronidase (10 mg/mL; Sigma-Aldrich, St. Louis, MO, USA) for 30 min at 37 °C and washed twice with phosphate-buffered saline (PBS). SM samples were rinsed twice with PBS, minced finely with sterilized scissors and digested using collagenase B (1 mg/mL; Roche Diagnostics, Rotkreuz, Switzerland) and bovine testicular hyaluronidase (2 mg/mL) at 37 °C for 2 h in RPMI-1640 culture medium supplemented with penicillin–streptomycin (10 μg/mL; Invitrogen, Carlsbad, CA, USA) and 5% fetal calf serum (FCS) (Biochrom AG, Berlin, Germany). To remove any undigested tissue the cell suspension was filtered through a 100-μm (BD Biosciences, San Jose, CA, USA) and a 40-μm pore-size cell strainer (EMD Millipore, Burlington, MA, USA). The filtered cell suspension was washed twice with PBS. Isolation of mononuclear cells (MNC) from PB, SF and SM samples was performed using Ficoll-Paque TM PLUS (GE Healthcare, USA) density gradient centrifugation. Subsequently, T cells were isolated from mononuclear cells using CD3 magnetic activated cell sorting (MACS) bead separation (Miltenyi Biotec, Bergisch Gladbach, Germany).

### 2.5. Flow Cytometry Analyses of Cell Surface Markers

To analyze CD4^+^ T cell surface marker expression in PB, SM and SF samples, multi-color flow cytometry was performed. Th subtypes were identified according to their preferential expression of surface markers using CXCR3, CCR5, CCR3, CCR4, CD161 and CCR6. Th1 cells were characterized using CXC3 and CCR5 labeling Th2 and Th17 cells were identified using CCR3 and CCR4 staining as well as labeling for CD161 and CCR6, respectively.

In brief, CD3^+^ MACS-isolated T cells from PB, SM and SF were washed twice in staining buffer, blocked with FcR blocking reagent (Miltenyi Biotec, Bergisch Gladbach, Germany) and then stained for 30 min at 4 °C with the following monoclonal antibodies (mAb)—Vioblue-labelled mAb against CD4 (clone VIT4, Miltenyi Biotec, Bergisch Gladbach, Germany), Alexa Fluor 488-labelled mAb against CXCR3 (clone 1C6), PE labelled mAb against CCR4 (clone 1G1), Alexa Fluor 647-labelled mAb against CCR3 (clone 2A3) and Allophycocyanin (APC) Cy7 labelled mAb against CCR5 (clone 2D7), fluorescein isothiocyanate (FITC)-labelled mAb against CD161 (clone DX12), PE-Cy7-labelled mAb against CCR6 (clone 11A9), PE labelled mAb against CCR4 (clone 1G1) and APC-Cy7-labelled mAb against CD4 (clone RPA-T4).

Before flow cytometric detection, 0.5 μg/mL 7-aminoactinomycin D (7-AAD) (eBioscience) was added to the cell suspensions to exclude cell debris and dead cells. Flow analysis was performed using a MACSQuant Analyzer (Miltenyi Biotec, Bergisch Gladbach, Germany). Data analysis was performed using FlowJo version 9.6 (FlowJo, Ashland, OR, USA). The cut-off for all cell surface markers was defined based on fluorescence minus one (FMO) controls, as described previously [13]. Antibodies and cell preparation solutions were purchased from BD Biosciences, USA, if not stated otherwise

### 2.6. Statistical Analyses

Descriptive statistics of demographic and clinical parameters are expressed as mean ± standard deviation (SD) and range. Descriptive data of the flow cytometry analyses are presented as mean ± standard error of the mean (SEM). To reveal differences in Th cell distribution between distinct tissue samples (PB, SM, SF) analysis of variance was performed. Due to the predominantly non-parametric distribution of Th cells Kruskal-Wallis test followed by Dunn’s multiple comparison test was used. Spearman’s rank correlation coefficient was performed to examine correlations between cell distribution data and clinical parameters (K&L score, NRS, OKS-12, AKSS, FFbH-OA). *p*-values < 0.05 were considered statistically significant. Prism version 6.01 software (GraphPad Software Inc., La Jolla, CA, USA) was used for statistical analysis.

## 3. Results

### 3.1. Clinical Characteristics of the Study Population

Sociodemographic and clinical data of the study population are presented in Table 1.

In brief, a total of 47 patients (74.5% female, 25.5% male) were included in this study. Thirty patients had BC OA, whereas 17 patients were diagnosed with UC OA. Mean age (±SD) was 67.5 (±8.7) years and body mass index (BMI, ±SD) was 29.8 (±6.2) kg/m^2^. K&L scores ranged from II-IV. The majority of all OA patients (59.6%) had a K&L score of 4, although only 11.8% of UC OA patients were graded K&L 4. Mean knee pain (±SD) was rated 7.2 (±2.1) and OKS-12 score 33.8 (±9.0). Mean AKSS knee and functional score was 42.8 (±15.2) and 56.6 (±21.5), respectively. Mean functional disability due to OA was rated 54.5 (±23.0).

### 3.2. T Cell Profile in in OA Joints and Peripheral Blood

To characterize the distribution of T cell subsets in different tissue types of OA patients flow cytometry of matching PB, SM and SF samples was performed. Overall flow cytometry data are presented in Table 2.

In brief, statistical analysis revealed significant differences in tissue specific T cell infiltration. Whereas concentration levels of CD3^+^ and CD4^+^ cells in PB and SM were significantly higher than in SF samples, no differences were observed comparing PB with SM cell concentration levels. For CXCR3^+^ and CCR5^+^ Th1 cells concentration levels differed significantly between all tissue types with highest levels in SM and lowest levels in PB samples. Similar to the distribution pattern of the total CD4^+^ Th population, concentration levels of Th2 cells labeled for CCR3 and CCR4 are significantly higher in PB and SM than in SF. Highest Th2 concentrations were also detected in SM samples. In contrast, Th17 cells (CD161^+^ and CCR6^+^) show highest concentration levels in PB.

**Figure 1:**
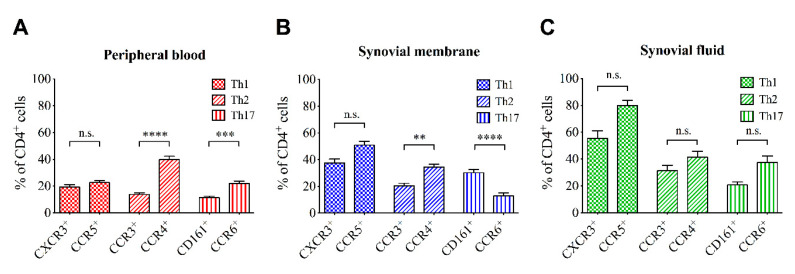
Percentage rates of different T helper cell subsets (Th1, Th2, Th17) within (**A**) peripheral blood, (**B**) synovial membrane and (**C**) synovial fluid samples according to their surface marker expression. *p*-Values < 0.05 were considered statistically significant and are indicated with asterisks: ** *p* < 0.01; *** *p* < 0.001; **** *p* < 0.0001. Data are presented as mean ± standard error of the mean (SEM). CD = cluster of differentiation; CXCR3 = C-X-C motif chemokine receptor 3; CCR = C-C chemokine receptor; n.s. = not significant.

Figure 1 displays the percentage rate of different Th subsets in PB, SM and SF samples according to their surface marker expression. Significant differences were observed between the percentage rates of Th subsets depending on the expressed surface markers in PB and SM but not SF. Interestingly, percentage rates of Th cells expressing surface markers for Th2 (CCR3 and CCR4) and Th17 (CD161 and CCR6) subsets were significantly different in PB and SM depending on the analyzed surface marker. In fact, the percentage rates of CCR4^+^ Th cells were significantly higher than of CCR3^+^ Th cells in both PB and SM although both label Th2 cells. Furthermore, the percentage rates of Th17 cells in PB and SM are significantly different depending on the expression of CD161 or CCR6. When compared to CCR6 expression, the percentage rate of CD161 expressing Th cells is significantly lower in PB. However, in SM the percentage rate of CD161^+^ Th cells is approximately twice as high as the percentage rate of CCR6^+^ Th cells. No differences were observed in Th1 cells expressing CXCR3 or CCR5 in all tissue types. Detailed information about the one-way ANOVA related results is outlined in Tables S1–S3. Between UC and BC OA no significant differences in the infiltration pattern were observed (see Table S4).

### 3.3. Correlation of Cell Infiltration and Clinical Parameters

To assess the clinical relevance of tissue specific T cell infiltration in knee OA correlation analyses between concentration levels of different Th subsets in PB, SM and SF samples and clinical parameters including OA-related knee pain and disability were performed.

#### 3.3.1. CD4^+^ Cell Infiltration and Clinical Parameters

**Figure 2:**
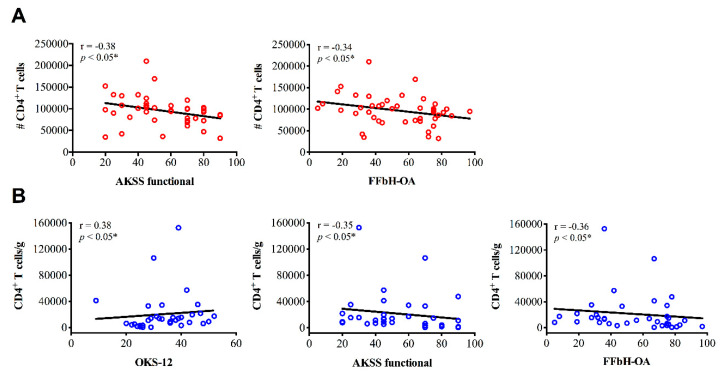
Correlation analyses between CD4^+^ T cells and clinical parameters of knee osteoarthritis (OA) patients. Spearman’s rank correlation coefficient (r) revealed significant moderate associations between CD4^+^ T cell infiltration in (**A**) peripheral blood (PB) and (**B**) synovial membrane (SM) samples of knee OA patients and joint function. (**A**) Higher numbers (#) of CD4^+^ T cells in PB correlate with poorer AKSS functional and FFbH-OA scores. (**B**) Increased CD4^+^ concentration levels in SM samples are associated with reduced knee function across all functional scores (OKS-12, AKSS functional, FFbH-OA). *p*-Values < 0.05 were considered statistically significant and are indicated with asterisks: * *p* < 0.05. AKSS = American Knee Society score; CD = cluster of differentiation; FFbH-OA = Hannover Functional Questionnaire of functional disability caused by OA; OKS-12 = Oxford Knee Score.

Spearman’s rank correlation coefficient was performed to examine correlations between CD4^+^ cell distribution and clinical parameters. Most importantly, the infiltration of SM with CD4^+^ cells was associated with greater OA-related disability. Across all functional scores (OKS-12, AKSS functional and FFbH-OA) increased concentration levels of CD4^+^ cells correlated significantly with poorer knee function. Additionally, higher numbers of CD4^+^ cells (total cell count) in PB samples were associated with decreased outcome in AKSS and FFbH-OA scores (Figure 2). Relevant correlations between CD4^+^ cells in SF samples and clinical parameters were not observed.

#### 3.3.2. Th1 Cell Infiltration and Clinical Parameters

**Figure 3:**
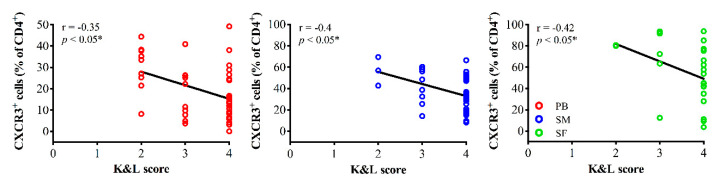
Correlation analyses between the mean percentage rate of CXCR3^+^ cells (% of CD4^+^ cells) and OA-severity. Higher percentage rates of CXCR3^+^ cells in peripheral blood (PB, red), synovial membrane (SM, blue) and synovial fluid (SF, green) were associated with less severe radiographic OA assessed using the Kellgren and Lawrence scoring system (K&L score). Correlation analyses were performed using Spearman’s rank correlation coefficient (r). CD = cluster of differentiation; CXCR3 = C-X-C motif chemokine receptor 3.

Interestingly, the mean percentage rate of CXCR3^+^ cells (% of CD4^+^) significantly correlated with K&L scores. Higher rates of CXCR3^+^ cells in all tissue types (PB, SM and SF) were observed in patients with less severe OA (Figure 3). In PB samples both increasing total CXCR3^+^ cell counts and concentration levels are associated with lower K&L scores, additionally.

**Figure 4:**
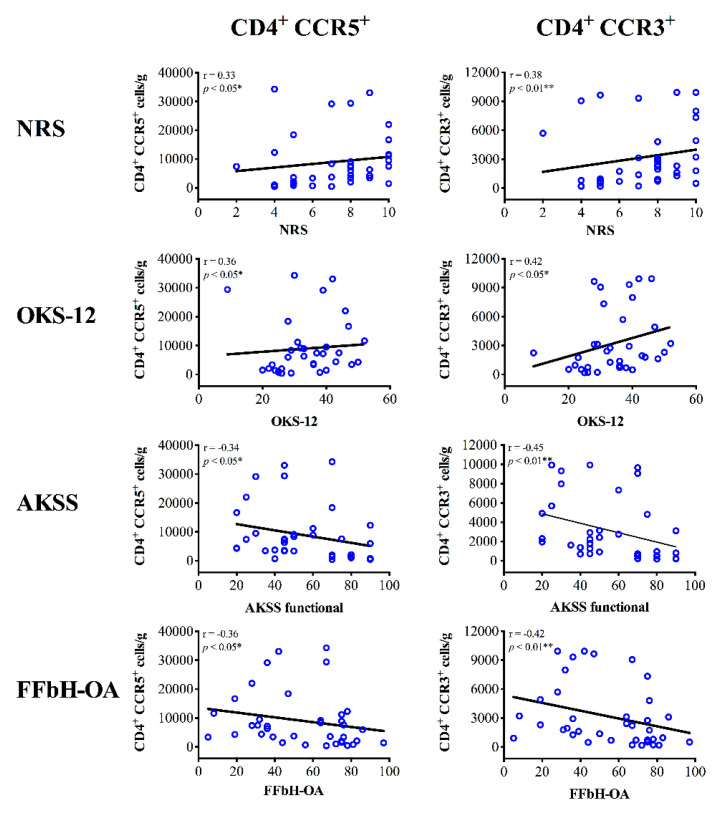
Correlation analyses between synovial membrane infiltration of Th1 and Th2 cells and clinical parameters of knee OA patients. Spearman’s rank correlation coefficient (r) revealed significant associations between Th1 (CD4^+^ CCR5^+^) and Th2 (CD4^+^ CCR3^+^) cell infiltration in the synovial membrane (SM) of knee OA patients and both joint pain and function. Increasing concentrations levels of CCR5^+^ and CCR3^+^ Th cells in SM samples of knee OA correlate with higher pain intensity (NRS) and poorer knee function (OKS-12, AKSS functional, FFbH-OA). *p*-Values < 0.05 were considered statistically significant and are indicated with asterisks: * *p* < 0.05, ** *p* < 0.01. AKSS = American Knee Society score; CCR = C-C chemokine receptor; CD = cluster of differentiation; FFbH-OA = Hannover Functional Questionnaire of functional disability caused by OA; NRS = numerical rating scale; OKS-12 = Oxford Knee Score.

In contrast, infiltration of CCR5^+^ Th cells in PB, SM and SF samples did not correlate with OA severity. In fact, patients with increasing concentration levels of CCR5^+^ Th cells in the SM show more severe joint pain (NRS) and greater disability across the applied functional scores (OKS-12, AKSS functional, FFbH-OA) (Figure 4). Associations between CCR5^+^ Th cell infiltration in PB or SF and clinical outcome have not been observed.

#### 3.3.3. Th2 Cell Infiltration and Clinical Parameters

Interestingly, CCR3^+^ Th cells show a similar correlation pattern when compared to the results for CCR5^+^ (Th1) cells (Figure 4). There were no associations between CCR3^+^ Th cell infiltration in PB and clinical outcome. Yet, infiltration of the SM with CCR3^+^ Th cells correlated significantly with OA-related knee pain and disability. As illustrated in Figure 4, OA patients with higher concentration levels of CCR3^+^ Th cells in SM samples reported to suffer from more severe pain (NRS) and reduced joint function in all functional assessment scores (OKS-12, AKSS functional, FFbH-OA). Furthermore, increasing numbers (total cell count) of CCR3^+^ Th cells in SF significantly correlate with poorer outcome in the AKSS functional score. In contrast, for CCR4^+^ Th cells correlation analyses only revealed associations between SM infiltration and the AKSS functional score. Higher concentration levels and numbers (total cell count) of CCR4^+^ Th cells in SM correlated with reduced AKSS functional scores (r = −0.34, * *p* < 0.05). Correlations between CCR4^+^ Th cells and other assessment scores were not observed neither in SM nor in samples of PB and SF.

#### 3.3.4. Th17 Cell Infiltration and Clinical Parameters

**Figure 5:**
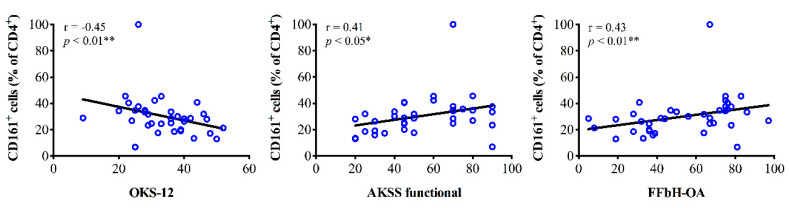
Correlation analyses between synovial membrane infiltration of CD161^+^ T helper cells (Th, CD4^+^) and clinical parameters of knee OA patients. Spearman’s rank correlation coefficient (r) revealed that the mean percentage rate of CD161^+^ Th cells (% of CD4^+^) significantly correlated with joint function. Higher percentage rates of CD161^+^ Th cells were associated with better outcome in OKS-12, AKSS functional and FFbH-OA scores. *p*-Values < 0.05 were considered statistically significant and are indicated with asterisks: * *p* < 0.05, ** *p* < 0.01. AKSS = American Knee Society score; CD = cluster of differentiation; FFbH-OA = Hannover Functional Questionnaire of functional disability caused by OA; OKS-12 = Oxford Knee Score.

In brief, there were no associations between clinical outcome scores and infiltration of PB for both CD161^+^ and CCR6^+^ Th cells. In contrast, analyses of SM samples revealed that the mean percentage rate of CD161^+^ Th cells (% of CD4^+^) significantly correlated with joint function. Higher percentage rates of CD161^+^ Th cells were associated with better outcome in OKS-12, AKSS functional and FFbH-OA scores (Figure 5). CD161^+^ Th cell concentration levels in SM did not show associations with clinical data. For CCR6^+^ Th cells we observed that higher concentration levels in SM and SF were associated with worse AKSS functional and OKS-12 scores, respectively.

## 4. Discussion

Even though the contribution of inflammatory processes in the pathogenesis of OA has become widely accepted, the OA-induced inflammatory pattern has never been evaluated in relation to the clinical symptoms of OA patients. Yet, a translational approach is of major importance to unravel clinically relevant inflammatory pathways and/or cellular targets in order to develop novel treatment strategies for OA. Especially, infiltration of mononuclear cells into OA joints and elevation of serum and synovial levels of inflammatory cytokines have been described to be associated with OA [6,13,24,25]. Immunohistological and flow cytometry studies have demonstrated that Th cells present one of the most prevalent types of activated inflammatory cells in the synovium [12,15,16] and are highly enriched in PB and SF samples of OA patients when compared to samples of age-matched healthy controls [18,19]. Although important advances in the characterization of OA-associated inflammatory processes have been made, the clinical relevance of these mechanisms remains elusive. In this study we provide an extensive analysis of the systemic and joint-associated Th cell distribution pattern in relation to OA-associated symptoms including joint pain and function. Our findings indicate that infiltration of the SM with Th cells might not only contribute to OA pathophysiology but also significantly to the clinical symptoms of OA. Compared to PB and SF, Th infiltration of the SM showed significant associations with clinical data across all Th subtypes. Only few correlations have been observed between Th cell infiltration of PB and SF and clinical outcome.

Interestingly, robust correlations between OA-related knee pain and disability (across all functional scores) and SM concentration levels of both CCR3^+^ and CCR5^+^ Th cells were detected. Both chemokine receptors can be expressed by different Th cell subsets, especially since surface marker expression varies during diapedesis [26]. CCR5 as well as CXCR3 are widely considered to be preferentially expressed by the Th1 subpopulation [27]. CCR5 is a crucial chemokine by exerting T cell recruitment to sites of inflammation but is also involved in its containment [28]. Diapedeses of T cells into inflamed SM or SF occurs by selective migration and CXCR3/CCR5-activating chemokines [29,30,31]. CD4^+^CCR5^+^ T cells were shown to have higher susceptibility to apoptosis in primary progressive multiple sclerosis and visceral leishmaniosis compared to CD4^+^CXCR3^+^ [32,33]. Further, CCR3^+^ T cells were shown to secrete Th2-specific cytokine IL-4, indicating a preferential expression in Th2 cells [34,35], although moderate and variable [36,37], whereas other groups could not validate this [27]. CCR3 mediates the effect of several chemokines and thereby facilitates migration and accumulation of inflammatory cells in inflamed tissue [38]. It was shown to be upregulated in OA synovium [39] in comparable amounts compared to other forms of arthritides [40,41], probably to effectuate CD4^+^ T cell and monocyte retention in the joint [42].

Due to the characteristic release of distinct cytokines, Th1 and Th2 responses were historically considered pro- and anti-inflammatory, respectively [43]. Our results indicate that both Th1 and Th2 polarization might be relevant to define end-stage OA on a cellular level. Although, studies have suggested that alterations of Th2 cells in OA are limited [17] synovial Th2 cell infiltration might be of greater importance as previously expected when including clinical data in the interpretation of cellular analyses. Basically, OA is predominantly triggered by pro-inflammatory processes. Nevertheless, our results indicate involvement of anti-inflammatory mechanisms since synovial Th2 cell infiltration is related to poorer joint function and greater pain in patients suffering from knee OA. We hypothesize that the correlation between synovial Th2 cell infiltration and worse clinical outcome represents a counteractive response of the immune system to the detrimental catabolic effects of pro-inflammatory mediators in order to restore or maintain the balance between pro- and anti-inflammatory pathways. In line with this, increasing levels of anti-inflammatory cytokines such as Interleukin (IL)-10 and IL-13 in SF samples from knee OA patients significantly correlate with OA-related symptoms as previously reported [6]. Besides IL-4 and Il-5, IL-10 and IL-13 are characteristic cytokines released by Th2 cells. Although a causal relationship between synovial Th2 cell infiltration and nociceptive or disabling effects seems unlikely, infiltrating CCR3^+^ T cells might serve as a marker of OA-related pain and disability by displaying the competition of anti- and pro-inflammatory processes in clinically relevant OA. In contrast, synovial infiltration of Th1 cells might be causally related to OA progression and symptoms. Th1 cells are characterized by releasing IL-2, interferon-γ (IFN-γ) and tumor necrosis factor α (TNFα) and accumulation of Th1 cells in both SM and SF has been reported in OA knee joints [14,44,45,46,47]. In line with this, high concentration levels of pro-inflammatory mediators such as IL-1β and TNFα haven been detected in in SF samples of OA patients when compared to healthy controls [48]. Pro-inflammatory mediators including IL-1β, TNFα, IL-6, IL-15, IL-17 and IL-18 are known to disrupt metabolic homeostasis by promoting catabolic processes and enzymatic cartilage degradation [49]. Furthermore, direct and indirect pro-nociceptive effects of pro-inflammatory cytokines have been described in both experimental models of pain and human OA [7,8,50,51,52,53]. Interestingly, our correlation analyses suggest that involvement of Th1 cells in OA progression and symptoms depends on the expression of different Th1 surface markers. Whereas higher percentage rates of CXCR3^+^ cells in all tissue types (PB, SM and SF) correlated with lower OA-stages, no associations between OA severity and CCR5^+^ cells have been observed. Instead, synovial CCR5^+^ cell infiltration was related to greater pain expression and poorer knee function. Recent studies indicate that the CCR5 serves as functional receptor for several cytokines [54,55] and is expressed in normal and OA chondrocytes [56]. Furthermore, CCR5 has been detected in synovial fluid and tissue of both OA and rheumatoid arthritis (RA) patients [40,57]. In experimental models of OA, CCR5 deficiency resulted in reduced cartilage degradation stressing the important role of CCR5 in the pathogenesis of OA. In a rat model of adjuvant-induced arthritis CCR5 expression on macrophages seems to maintain joint inflammation [58]. In contrast, inhibiting CCR5 mediated signaling ameliorates articular destruction and signs of inflammation [59,60]. In human RA accumulation of CCR5^+^ Th cells into inflamed joints has been observed [61]. To our knowledge, this is the first study establishing a link between CCR5^+^ Th cells and OA-related pain and disability although a cross-talk between pain perception and CCR5 signaling has been described [62,63]. Potentially, accumulation of CCR5^+^ Th cells in the synovium might trigger joint inflammation via increases of pro-inflammatory mediators such as TNF and IFN-γ which could result in cytokine-induced sensitization of peripheral nociceptors and pain. Indeed, IL-6 and TNFα lead to peripheral sensitization of joint nociceptors in experimental models of OA [64,65] and release of IL-6 from human OA synovial fibroblasts has been reported [66]. Surprisingly, no associations between OA-related symptoms and infiltration of CXCR3^+^ Th cells were observed. Instead, CXCR3^+^ Th cell infiltration seems to be inversely related to OA-severity independent of the tissue type. Higher percentage rates of CXCR3^+^ cells (% of CD4^+^ cells) in PB, SM and SF samples from OA patients correlated significantly with lower K&L scores. This indicates that CXCR3 expression on Th cells might drive OA-progression in early stages of the disease. CXCR3 is primarily expressed on activated T and Natural Killer (NK) cells. In a collagenase-induced OA model mobilization of immune cells from bone marrow has been reported in early OA phases and CXCR3 expression promotes articular accumulation of NK cells and macrophages. Importantly, CXCR3 knock-out prevented OA development [67]. Although the underlying mechanisms differ between experimentally induced OA using collagenase and human OA, these findings suggest an important role of CXCR3 expression on mononuclear cells in early phases of the disease and therefore support our results. High percentages of CXCR3^+^ Th cells might reflect increasing inflammatory responses. Since the percentage rate of CXCR3^+^ Th cells was highest in patients with mild radiographic OA we suggest that inflammation is most abundant in early disease stages and triggers OA progression.

Controversial findings have been reported regarding the Th17 profile in OA patients. Whereas early investigations suggested little alterations of the Th17 profile in PB samples of patients with OA, more recent studies found significantly higher numbers of circulating Th17 cells when compared to healthy controls. In contrast to Th1 and Th2 cells, we observed highest Th17 concentration levels in PB and not in SM samples independent of the surface marker (CD161, CCR6) [17,68]. Nevertheless, correlation analyses only revealed associations between synovial Th17 cells and clinical parameters. Interestingly, higher percentage rates of synovial CD161^+^ Th cells correlated with better joint function whereas increasing concentration levels of CCR6^+^ cells in SM and SF samples were associated with worse functional outcome. On the one hand, these conflicting results might indicate that different surface markers expressed on the same cell type could mediate distinct functional responses. On the other hand, the controversial results might display the methodological challenge of using surface markers to identify different subsets of Th cells. Expression of the applied Th surface markers is not exclusively restricted to a specific Th subset. For example, CD161 expression was also found to a distinct subpopulation of regulatory T cells (Tregs) displaying high proinflammatory potential despite presenting classic Treg signatures (FoxP3+ CD4+) [69]. Thus, surface marker expression helps to characterize but does not define a specific Th subset. Therefore, the examined Th subpopulations might co-express surface markers that predominantly label other Th subtypes [70]. This in turn affects the interpretation of our data. Further investigations using both intracellular and surface markers are needed to conclusively identify the different Th subsets. Nevertheless, using surface marker expression for Th subset analyses provides an adequate approximation of the Th cell infiltration pattern as well as the opportunity to uncover receptors of potential clinical relevance in the pathogenesis of OA.

Of note, cell infiltration data might be influenced by sociodemographic and clinical parameters such as age, gender, BMI and joint inflammation. Adjustment to possible confounding factors was not performed in our analysis. Although OA severity ranged from K&L scores II to IV, all patients suffered from clinically advanced OA and therefore underwent surgery. Thus, the reported Th cell infiltration pattern represents the inflammatory status of patients requiring surgery due to OA-induced symptoms and/or functional limitations. Yet, generalization of our findings needs to be performed carefully as early and intermediate OA stages might present a different Th cell infiltration profile. Variability across patients was not tested in detail but might affect the interpretation of the results.

Further clinical trials using intracellular and surface expression markers for Th analysis are needed to conclusively untangle the role of the Th subsets in the pathogenesis of OA-related symptoms. For this purpose, our results provide a solid foundation.

## 5. Conclusions

In conclusion, the current study provides an extensive profile of the Th cell infiltration pattern in PB, SF and SM from patients with clinically relevant knee OA. Th cell infiltration of the SM might play a crucial role not only in the pathogenesis of OA but also in the development of OA-related knee pain and disability.

## Figures and Tables

**Table 1:** Study population.

	Total Study Population	UC OA	BC OA
Number of patients, *n*	47	17	30
Gender, *n* (%)			
Male	12 (25.5%)	3 (17.6%)	9 (30.0%)
Female	35 (74.5%)	14 (82.4%)	21 (70.0%)
Age, years	67.5 ± 8.7 (47–83)	65.4 ± 8.1 (49–76)	68.7 ± 9 (47–83)
BMI (kg/m^2^)	29.8 ± 6.2 (20.3–50.1)	30.0 ± 7.1 (20.8–46.1)	29.7 ± 5.8 (20.3–50.1)
K&L score, *n* (%)			
2	9 (19.1%)	8 (47.1%)	1 (3.3%)
3	10 (21.3%)	7 (41.2%)	3 (10.0%)
4	28 (59.6%)	2 (11.8%)	26 (86.7%)
Knee pain, NRS (0–10)	7.2 ± 2.1 (2.0–10.0)	6.9 ± 1.6 (4.0–10.0)	7.3 ± 2.3 (2.0–10.0)
OKS-12 (Pt.)	33.8 ± 9.0 (9.0–52.00)	34.5 ± 4.5 (26.0–43.0)	34.6 ± 10 (9.0–52.00)
AKSS			
Knee score (Pt.)	42.8 ± 15.2 (14.0–73.0)	48.5 ± 14.5 (18.0–73.0)	39.4 ± 14.9 (14.0–70.0)
Functional score (Pt.)	56.6 ± 21.5 (20.0–90.0)	62.8 ± 22.9 (20.0–90.0)	53.0 ± 20.2 (20.0–90.0)
FFbH-OA (%)	54.5 ± 23.0 (5.0–97.0)	60.9 ± 19.0 (28.0–86.0)	50.8 ± 24.6 (5.0–97.0)

Sociodemographic and clinical parameters of the study population are displayed. Data are presented as mean ± standard deviation (SD; range). OA = osteoarthritis; UC = unicompartmental; BC = bicompartmental; BMI = body mass index; K&L score = Kellgren and Lawrence score; NRS = numerical rating scale; OKS-12 = Oxford Knee Score; AKSS = American Knee Society Score; FFbH-OA = Hannover Functional Questionnaire of functional disability caused by OA.

**Table 2:** T cell infiltration in peripheral blood (PB), synovial membrane (SM) and synovial fluid (SF).

T Cells	PB	SM	SF	*p*-Value		
	Mean ± SEM	Mean ± SEM	Mean ± SEM	PB:SM	PB:SF	SM:SF
**CD3^+^**						
Cell count	122,996 ± 5907	76,883 ± 16,445	15,553 ± 4448	**** <0.0001	**** <0.0001	** <0.01
Cells/mL (g)	15,249 ± 960.1	28,243 ± 6052	2026 ± 450.1	n.s.	**** <0.0001	**** <0.0001
**CD4^+^**						
Cell count	96,798 ± 5141	58,010 ± 13,322	7453 ± 2626	**** <0.0001	**** <0.0001	*** <0.001
Cells/mL (g)	12,034 ± 832.7	21,301 ± 4935	820.7 ± 218.3	n.s.	**** <0.0001	**** <0.0001
% of CD3^+^ cells	78.45 ± 1.39	71.64 ± 1.66	42.03± 3.3	* <0.05	**** <0.0001	**** <0.0001
**Th1**						
CD4^+^ CXCR3^+^						
Cell count	18,562 ± 2153	19,372 ± 3846	3879 ± 1499	n.s.	**** <0.0001	**** <0.0001
Cells/mL (g)	2449 ± 336.8	7253 ± 1484	442.6 ± 147.7	* <0.05	**** <0.0001	**** <0.0001
% of CD4^+^ cells	19.26 ± 1.88	37.57 ± 2.93	55.34 ± 5.73	*** <0.001	**** <0.0001	n.s.
CD4^+^ CCR5^+^						
Cell count	21,256 ± 1603	25,224 ± 4756	6757 ± 2499	n.s.	**** <0.0001	*** <0.001
Cells/mL (g)	2624 ± 220.0	9041 ± 1553	709.9 ± 202.6	** <0.01	**** <0.0001	**** <0.0001
% of CD4^+^ cells	22.67 ± 1.5	50.90 ± 2.81	79.77 ± 4.03	**** <0.0001	**** <0.0001	** <0.01
CD4+ CXCR3+ CCR5+						
Cell count	1683 ± 317.0	2055 ± 552.5	92.68 ± 18.15	n.s.	**** <0.0001	**** <0.0001
Cells/mL (g)	199.8 ± 37.7	836.2 ± 276.3	12.50 ± 3.4	* <0.05	**** <0.0001	**** <0.0001
% of CD4^+^ cells	1.61 ± 0.27	5.11 ± 1.43	4.76 ± 1.50	**** <0.0001	n.s.	n.s.
**Th2**						
CD4^+^ CCR3^+^						
Cell count	13,223 ± 1253	8880 ± 1586	2052 ± 862.6	* <0.05	**** <0.0001	*** <0.001
Cells/mL (g)	1655 ± 182.3	3181 ± 523.5	208.1 ± 59.13	n.s.	**** <0.0001	**** <0.0001
% of CD4^+^ cells	13.88 ± 1.07	20.37 ± 1.96	31.34 ± 4.10	* <0.05	**** <0.0001	n.s.
CD4^+^ CCR4^+^						
Cell count	37,538 ± 3002	17,255 ± 3635	2080 ± 633	*** <0.001	**** <0.0001	*** <0.001
Cells/mL (g)	4738 ± 503.8	6310 ± 1371	239.8 ± 53.83	n.s.	**** <0.0001	**** <0.0001
% of CD4^+^ cells	39.80 ± 2.54	34.35 ± 2.25	41.40 ± 4.49	n.s.	n.s.	n.s.
CD4^+^ CCR3^+^ CCR4^+^						
Cell count	1635 ± 146.6	1206 ± 303.1	182.8 ± 38.4	*** <0.001	**** <0.0001	*** <0.001
Cells/mL (g)	204.5 ± 21.1	425.3 ± 101.7	25.07 ± 6.02	n.s.	**** <0.0001	**** <0.0001
% of CD4^+^ cells	1.74 ± 0.14	2.27 ± 0.38	8.62 ± 2.46	n.s.	*** <0.001	** <0.01
**Th17**						
CD4^+^ CD161^+^						
Cell count	9008 ± 708.4	5479 ± 1529	1132 ± 468.2	*** <0.001	**** <0.0001	** <0.01
Cells/mL (g)	1288 ± 128.6	882.7 ± 248.7	131 ± 93.14	** <0.01	**** <0.0001	** <0.01
% of CD4^+^ cells	11.42 ± 1.13	30.22 ± 2.41	20.73 ± 2.22	**** <0.0001	*** <0.001	n.s.
CD4^+^ CCR6^+^						
Cell count	20,035 ± 2189	1937 ± 574.5	3203 ± 1757	**** <0.0001	**** <0.0001	n.s.
Cells/mL (g)	2844 ± 385.7	334.9 ± 112.6	211.8 ± 134.9	**** <0.0001	**** <0.0001	n.s.
% of CD4^+^ cells	21.98 ± 1.83	12.79 ± 2.36	37.49 ± 4.86	** <0.01	n.s.	**** <0.0001
CD4^+^ CD161^+^ CCR6^+^						
Cell count	8128 ± 840.7	2253 ± 723.1	761.5 ± 353.3	**** <0.0001	**** <0.0001	n.s.
Cells/mL (g)	1200 ± 161.4	329.3 ± 89.58	91.90 ± 71.78	*** <0.001	**** <0.0001	n.s.
% of CD4^+^ cells	8.49 ± 0.68	11.16 ± 0.7551	10.92 ± 1.49	n.s.	n.s.	n.s.

The distribution of T cells in matching peripheral blood (PB), synovial membrane (SM) and synovial fluid (SF) samples of knee OA patients was assessed using flow cytometry. CD3^+^ MACS-isolated T lymphocytes were stained for CD4 to detect T helper cells (Th; CD3^+^CD4^+^, in Table referred to as CD4^+^). To analyze Th subpopulations (Th1, Th2, Th17) CD4^+^ cells were stained for distinct surface markers (Th1: CXCR3, CCR5; Th2: CCR3, CCR4; Th17: CD161, CCR6). For CD3^+^ MACS-isolated T lymphocytes mean cell count and concentration levels (cells/sample volume (mL) or weight (g)) are shown. For CD4^+^ cells the mean CD4^+^ T cell percentage rate (% of CD3^+^ cells stained positive for CD4) was calculated additionally. For different Th1, Th2 and Th17 cells total cell count, concentration levels and mean percentage rate of CD4^+^ cells are displayed. Kruskal-Wallis test followed by Dunn’s multiple comparison revealed significant differences in tissue infiltration for all Th cells. *p*-Values < 0.05 were considered statistically significant and are indicated with asterisks: * *p* < 0.05; ** *p* < 0.01; *** *p* < 0.001; **** *p* < 0.0001. Data are presented as mean ± standard error of the mean (SEM). CD = cluster of differentiation; CXCR3 = C-X-C motif chemokine receptor 3; CCR = C-C chemokine receptor; n.s. = not significant.

## Supplementary Materials

The following are available online at https://www.mdpi.com/2077-0383/9/8/2423/s1, Table S1: Results of the one-way ANOVA (Kruskal-Wallis test) followed by Dunn’s multiple comparison between the percentage rates of different T helper cell subsets in the peripheral blood; Table S2: Results of the one-way ANOVA (Kruskal-Wallis test) followed by Dunn’s multiple comparison between the percentage rates of different T helper cell subsets in the synovial membrane; Table S3: Results of the one-way ANOVA (Kruskal-Wallis test) followed by Dunn’s multiple comparison between the percentage rates of different T helper cell subsets in the synovial fluid; Table S4: T cell infiltration in PB, SM and SF depending on UC and BC OA.
