# Supplementary material for: T Helper Cell Infiltration in Osteoarthritis-Related Knee Pain and Disability
Source: J Clin Med. 2020 Jul 29;9(8):2423. doi: 10.3390/jcm9082423 (PMC7464429; doi:10.3390/jcm9082423)
Supplement: Supplementary file 1 [file jcm-09-02423-s001.pdf]

Table S1. Results of the one-way ANOVA (Kruskal-Wallis test) followed by Dunn's multiple comparison between the percentage rates of different T helper cell subsets in the peripheral blood.

Kruskal-Wallis Test	Dunn's Multiple Comparisons Test	Mean Rank Difference	*p*-Value
p-value < 0.0001	$CXCR3^+$ vs. $CCR5^+$	-25.58	>0.9999
	$CXCR3^+$ vs. $CCR3^+$	30.12	0.9204
	$CXCR3^+$ vs. $CCR4^+$	-85.58	<0.0001
	$CXCR3^+$ vs. $CD161^+$	50.95	0.0265
	$CXCR3^+$ vs. $CCR6^+$	-21.30	>0.9999
	$CCR5^+$ vs. $CCR3^+$	55.70	0.0082
	$CCR5^+$ vs. $CCR4^+$	-60.00	0.0029
	$CCR5^+$ vs. $CD161^+$	76.52	<0.0001
	$CCR5^+$ vs. $CCR6^+$	4.280	>0.9999
	$CCR3^+$ vs. $CCR4^+$	-115.7	<0.0001
	$CCR3^+$ vs. $CD161^+$	20.82	>0.9999
	$CCR3^+$ vs. $CCR6^+$	-51.42	0.0273
	$CCR4^+$ vs. $CD161^+$	136.5	<0.0001
	$CCR4^+$ vs. $CCR6^+$	64.28	0.0015
	$CD161^+$ vs. $CCR6^+$	-72.24	0.0002

ANOVA = analysis of variance; CD = cluster of differentiation; CXCR3 = C-X-C motif chemokine receptor 3; CCR = C-C chemokine receptor.

Table S2. Results of the one-way ANOVA (Kruskal-Wallis test) followed by Dunn's multiple comparison between the percentage rates of different T helper cell subsets in the synovial membrane.

Kruskal-Wallis Test	Dunn's Multiple Comparisons Test	Mean Rank Difference	*p*-Value
p-value < 0.0001	$CXCR3^+$ vs. $CCR5^+$	-38.61	0.1564
	$CXCR3^+$ vs. $CCR3^+$	61.01	0.0008
	$CXCR3^+$ vs. $CCR4^+$	6.689	>0.9999
	$CXCR3^+$ vs. $CD161^+$	23.77	>0.9999
	$CXCR3^+$ vs. $CCR6^+$	95.75	<0.0001
	$CCR5^+$ vs. $CCR3^+$	99.62	<0.0001
	$CCR5^+$ vs. $CCR4^+$	45.30	0.0399
	$CCR5^+$ vs. $CD161^+$	62.37	0.0005
	$CCR5^+$ vs. $CCR6^+$	134.4	<0.0001
	$CCR3^+$ vs. $CCR4^+$	-54.32	0.0047
	$CCR3^+$ vs. $CD161^+$	-37.25	0.1929
	$CCR3^+$ vs. $CCR6^+$	34.74	0.3050
	$CCR4^+$ vs. $CD161^+$	17.08	>0.9999
	$CCR4^+$ vs. $CCR6^+$	89.06	<0.0001
	$CD161^+$ vs. $CCR6^+$	71.99	<0.0001

CD = cluster of differentiation; CXCR3 = C-X-C motif chemokine receptor 3; CCR = C-C chemokine receptor.

Table S3. Results of the one-way ANOVA (Kruskal-Wallis test) followed by Dunn's multiple comparison between the percentage rates of different T helper cell subsets in the synovial fluid.

Kruskal-Wallis Test	Dunn's Multiple Comparisons Test	Mean Rank Difference	*p*-Value
p-value < 0.0001	$CXCR3^+$ vs. $CCR5^+$	-30.44	0.1560
	$CXCR3^+$ vs. $CCR3^+$	33.60	0.0701
	$CXCR3^+$ vs. $CCR4^+$	16.17	> 0.9999
	$CXCR3^+$ vs. $CD161^+$	53.29	0.0001
	$CXCR3^+$ vs. $CCR6^+$	21.46	> 0.9999
	$CCR5^+$ vs. $CCR3^+$	64.04	< 0.0001
	$CCR5^+$ vs. $CCR4^+$	46.60	0.0013
	$CCR5^+$ vs. $CD161^+$	83.73	< 0.0001
	$CCR5^+$ vs. $CCR6^+$	51.90	0.0002
	$CCR3^+$ vs. $CCR4^+$	-17.44	> 0.9999
	$CCR3^+$ vs. $CD161^+$	19.68	> 0.9999
	$CCR3^+$ vs. $CCR6^+$	-12.14	> 0.9999
	$CCR4^+$ vs. $CD161^+$	37.12	0.0299
	$CCR4^+$ vs. $CCR6^+$	5.295	> 0.9999
	$CD161^+$ vs. $CCR6^+$	-31.83	0.1309

CD = cluster of differentiation; CXCR3 = C-X-C motif chemokine receptor 3; CCR = C-C chemokine receptor.

Table S4. T cell infiltration in PB, SM and SF depending on UC and BC OA.

T cells	PB			SM			SF		
	UC<br>Mean ± SEM	BC<br>Mean ± SEM	UC:BC<br>*p*-Value	UC<br>Mean ± SEM	BC<br>Mean ± SEM	UC:BC<br>*p*-Value	UC<br>Mean ± SEM	BC<br>Mean ± SEM	UC:BC<br>*p*-Value
**$CD3^+$**									
Cell count	121988 ± 8867	123552 ± 7862	n.s.	47716 ± 14225	89224 ± 22348	n.s.	12508 ± 3805	16806 ± 6131	n.s.
Cells/mL (g)	13957 ± 1378	15962 ± 1279	n.s.	18927 ± 5769	32184 ± 8206	n.s.	2690 ± 1117	1752 ± 450	n.s.
**$CD4^+$**									
Cell count	98189 ± 7204	96031 ± 7003	n.s.	33582 ± 10439	68345 ± 18189	n.s.	3940 ± 1405	8900 ± 3638	n.s.
Cells/mL (g)	11256 ± 1120	12462 ± 1141	n.s.	13491 ± 4474	24606 ± 6711	n.s.	881.8 ±	795.6 ± 251.4	n.s.
% of $CD3^+$ cells	80.88 ± 1.97	77.10 ± 1.85	n.s.	65.92 ± 4.0	74.07 ± 1.47	*<0.05	36.41 ± 6.77	44.35 ± 3.72	n.s.
**Th1**									
$CD4^+$ $CXCR3^+$<br>% of $CD4^+$ cells	23.94 ± 3.42	±<br>16.67 ± 2.13	n.s.	45.18 ± 5.35	34.35 ± 3.37	n.s.	64.94 ± 9.91	51.39 ± 6.93	n.s
$CD4^+$ $CCR5^+$<br>% of $CD4^+$ cells	22.38 ± 2.57	22.83 ± 1.87	n.s.	56.29 ± 3.92	48.62 ± 3.59	n.s.	82.57 ± 7.36	78.61 ± 4.93	n.s
CD4+ CXCR3+ CCR5+<br>% of $CD4^+$ cells	1.50 ± 0.35	1.67 ± 0.38	n.s.	8.15 ± 4.5	3.82 ± 0.74	n.s.	4.08 ± 2.4	5.06 ± 1.93	n.s.
**Th2**									
$CD4^+$ $CCR3^+$<br>% of $CD4^+$ cells	15.24 ± 2.03	13.12 ± 1.23	n.s.	22.32 ± 3.06	19.55 ± 2.49	n.s.	39.76 ± 11.61	27.88 ± 3.28	n.s.
$CD4^+$ $CCR4^+$<br>% of $CD4^+$ cells	41.83 ± 4.11	38.68 ± 3.26	n.s.	35.13 ± 5.55	34.02 ± 2.28	n.s.	42.54 ± 10.49	40.93 ± 4.89	n.s.
$CD4^+$ $CCR3^+$ $CCR4^+$<br>% of $CD4^+$ cells	1.55 ± 0.17	±<br>1.85 ± 0.2	n.s.	1.38 ± 0.16	2.70 ± 0.52	n.s.	7.6 ± 3.57	9.07 ± 3.23	n.s.
**Th17**									
$CD4^+$ $CD161^+$<br>% of $CD4^+$ cells	11.24 ± 1.23	11.51 ± 1.59	n.s.	33.90 ± 7.41	28.61 ± 1.72	n.s.	25.36 ± 6.2	18.71 ± 1.65	n.s.
$CD4^+$ $CCR6^+$<br>% of $CD4^+$ cells	19.33 ± 3.41	23.20 ± 2.17	n.s.	16.91 ± 7.63	11.12 ± 1.29	n.s.	44.29 ± 11.45	34.51 ± 4.99	n.s.

$CD4^+$ $CD161^+$ $CCR6^+$ % of $CD4^+$ cells	8.15 ± 1.02	8.98 ± 0.88	n.s.	13.24 ±	10.30 ± 0.48	n.s.	12.03 ± 3.01	12.73 ± 1.59	n.s.

The distribution of T cells in peripheral blood (PB), synovial membrane (SM) and synovial fluid (SF) samples compared between unicompartmental (UC) and bicompartmental osteoarthritis (OA). T lymphocytes were stained for CD4 to detect T helper cells (Th; CD3+CD4+, in Table referred to as CD4+). To analyze Th subpopulations (Th1, Th2, Th17) CD4+ cells were stained for distinct surface markers (Th1: CXCR3, CCR5; Th2: CCR3, CCR4; Th17: CD161, CCR6). For CD3+ MACS-isolated T lymphocytes mean cell count and concentration levels (cells/sample volume (mL) or weight (g)) are shown. For CD4+ cells the mean CD4+ T cell percentage rate (% of CD3+ cells stained positive for CD4) was calculated additionally. For different Th1, Th2 and Th17 cells mean percentage rates of CD4+ cells (% of % of CD4+ cells) are displayed. Unpaired t-test was used to compare the mean percentage rates between UC and BC patients. *p*-Values < 0.05 were considered statistically significant and are indicated with asterisks: * $p < 0.05$. Data are presented as mean ± standard error of the mean (SEM). CD = cluster of differentiation; CXCR3 = C-X-C motif chemokine receptor 3; CCR = C-C chemokine receptor; n.s. = not significant.
